# ‘Everybody Likes a Drink. Nobody Likes a Drunk’. Alcohol, Health Education and the Public in 1970s Britain

**DOI:** 10.1093/shm/hkw094

**Published:** 2016-12-22

**Authors:** Alex Mold

**Keywords:** public health, alcohol, health education, health promotion

## Abstract

This article examines the development of alcohol health education in Britain during the 1970s, using this as a way to explore the nature of public health and the place of the public within it. Focusing on a set of local health education campaigns, an expert committee report on alcohol prevention and a public consultation exercise on alcohol, the article highlights the presence of three different ‘publics’. Health education campaigns tended to focus on the individual drinker, but the drinking habits of the whole population were also of concern. So too were the rights and responsibilities of citizen-consumers. These three publics—drinkers, the population and citizen-consumers—were often in conflict with one another, and though it was drinkers that became the object of alcohol policy, the needs of the population, and of citizen-consumers, could not be ignored.

In 1974, in the north east of England, the Health Education Council (HEC) launched a pilot programme to increase public awareness about alcohol problems. Under the tag line ‘Everybody likes a drink. Nobody likes a drunk.’ the campaign made use of billboard posters, advertisements in local newspapers and television commercials in order to encourage people in the region to ‘Drink in moderation’ and not ‘let alcohol go to your head’. The campaign, designed by the well-known advertising agency Saatchi and Saatchi, reached a large proportion of the local population. Around 90 per cent of people surveyed said that they had watched the television advertisements, and 60 per cent of respondents recalled seeing the campaign posters.^1^ Local alcohol treatment and advice services were inundated with people seeking help for alcohol problems, a sign, the HEC believed, of the initiative’s success.^2^

Not everyone involved in the campaign, however, was so sure. On the ground, service providers felt that they had not been consulted sufficiently about the campaign and that it was insensitive to local needs. The programme’s methods were also unpopular, with the slogan ‘Everybody likes a drink. Nobody likes a drunk.’ attracting particular opprobrium. Criticism of the campaign’s tactics was underpinned by more fundamental misgivings about the approach taken. There was little consensus about what it meant to ‘drink in moderation’ and whether or not a sceptical public could be convinced to consume alcohol ‘sensibly’. Some experts, such as the leading addiction researcher Professor Griffith Edwards, argued that health education simply would not work, at least not alone. Instead, Edwards suggested, more should be done to limit drinking at the societal level, as the number of people with alcohol problems was related to the overall amount of alcohol consumed by the population.^3^ Making sure that the price of alcohol did not decrease, and ensuring that adequate controls on the availability of drink remained in place, he suggested, would be more effective than health education in dealing with alcohol problems.

Conflict over how to respond to alcohol related issues points to more fundamental difficulties faced by public health and its dealings with the public in this period. By examining the approach taken to alcohol health education in the 1970s and early 1980s, this article aims to tease out larger issues surrounding the nature of public health in Britain and the place of the public within it. The apparent rise of the incidence of chronic disease linked with individual behaviour, such as smoking and lung cancer, posed new challenges to public health policy makers. Could the public be persuaded to change their behaviour, or did the environment need to be altered? Should public health initiatives target individuals, or the entire population? How far should public health intervene in citizens’ lives?

Attempts to find answers to such questions bedevilled the response to alcohol as a public health problem. To explore and explain these in more detail, this article will begin by considering the history of health education and responses to alcohol. Neither health education nor alcohol problems were ‘new’, but the nature of the issue and the ways in which it was responded to took on a particular character in the 1970s. This can be seen in three areas. The first concerns a series of alcohol education campaigns designed and implemented by the HEC in the north east of England. The ‘Everybody likes a drink. Nobody likes a drunk.’ campaign was the first of three different attempts to educate the public about the dangers of alcohol that took place between 1974 and 1981. Looking at each of these campaigns points to an evolution in health education’s tactics, targets and techniques, but also to problems with these on both a practical and a more fundamental level. Some policy makers and practitioners had doubts about the ability of health education to address alcohol problems. Such reservations are explored in more detail in the second area of focus: an analysis of the making of the Advisory Council on Alcohol’s (ACA) report on *Prevention*, published in 1977.^4^ The ACA attempted to resolve tension between those who believed that health education could prevent the development of alcohol problems and those who argued that it was ineffective; that more emphasis should instead be placed on increasing the price of alcohol and reducing its availability. In their final report the ACA concluded that such issues demanded wider public discussion, and they recommended that a public consultation on alcohol be initiated. The long gestation of the resulting discussion document, *Drinking Sensibly*, forms the third area of focus. The production of *Drinking Sensibly* between 1977 and 1981 was hindered by considerable inter-departmental conflicts. Officials in the Department of Health and Social Security (DHSS) wanted to endorse the population level approach to dealing with alcohol problems, but other departments, such as the Treasury and the Ministry of Agriculture, Fisheries and Food (MAFF), were reluctant to support a policy that might result in lowering the tax revenue from alcohol. As a result, *Drinking Sensibly* rejected population level measures like increasing the duty on alcohol, and instead recommended a policy of more health education based on the concept of ‘drinking sensibly’.^5^

In some ways this could be seen as a victory for the health education approach, as efforts to turn Britain into a nation of ‘sensible drinkers’ became the cornerstone of alcohol policy. The ‘sensible drinker’ chimed perfectly with elements of the ‘new public health’ that focused on getting individuals to take responsibility for their own health and limiting the risk that they posed to others through preventive actions.^6^ Yet, as this article will demonstrate, the ‘sensible drinker’ was just one of the various publics at work within alcohol education and public health more broadly. Alongside a focus on the individual drinker, the drinking habits of the population were also of concern. So too were the rights and responsibilities of citizen-consumers. These three publics—drinkers, the population and citizen-consumers—were often in conflict with one another, and though it was drinkers that became the object of alcohol policy, the needs of the population, and of citizens-consumers, could not be ignored.

## Three Publics

Confusion about the nature of public health and the kinds of publics it involved was not a new problem, but it became particularly acute in the latter half of the twentieth century.^7^ As David Cantor has observed, there was no ‘general public’, only ways of seeing it. Cantor suggests that up until the 1930s, the public were regarded as a largely undifferentiated mass, but after this period the public began to fragment. The establishment of the National Health Service, the development of consumerism and the application of epidemiological categories began to break up the general public into different groups. By the 1970s, Cantor contends, ‘the notion of an undifferentiated public was much harder to sustain, and differences, which might once have been portrayed as variations within the mass general public, came to be marks of different publics’.^8^ The nature of these various publics and their place within post-war public health requires further exploration, but by pointing to three distinct but overlapping publics—the individual drinker; the population and the citizen-consumer—this article will begin to open up the categories of the public and public health to analysis.^9^

On the surface, individual drinkers would seem like the easiest group to define—anyone who consumed alcohol was a ‘drinker’—but even here there were considerable variations. How much and how frequently an individual drank, his or her relationship to alcohol, and the consequences of alcohol use for that individual and for society all played a part in shaping different categories of drinker. ‘Alcoholics’, ‘problem drinkers’, ‘heavy drinkers’ and ‘alcohol misusers’ had long been of interest to both the state and medical professionals, but in the 1960s and 1970s the ‘moderate’ or ‘sensible’ drinker also became an object of concern. This was related to two developments. First, individual behaviour was increasingly seen as a cause of public health problems. As will be discussed in greater detail below, the linking of behaviours such as smoking to diseases like lung cancer meant that individuals and their lifestyles were of concern to public health. Secondly, the application of epidemiology to this new category of behaviour-related conditions broadened the scope of public health to encompass the whole population as well as ‘risky’ individuals. The focus shifted from ‘problem’ drinkers of one sort or another to include all drinkers.^10^

This interest in the epidemiology of alcohol use and other lifestyle issues conjured another public into being: the population. This was not, of course, a new concept—the accumulation of data about the populace was a crucial part of modern state formation—but post-war epidemiology constructed the population and its relationship to the individual and the environment in ways that had a profound impact on the development of public health policy and practice. A key figure in British public health during this period, the epidemiologist Jerry Morris, defined epidemiology as ‘the study of health and disease of populations and of groups in relation to their environment and their ways of living.’^11^ Individuals, their behaviour and their environment, were both part of the population and distinct from it. As Nancy Krieger notes, ‘individual’ and ‘population’ were not antonyms.^12^ Nonetheless, a population orientated view of public health problems could result in a change of emphasis and thus a change of policy. A population level approach to alcohol problems could be found in a thesis first put forward in 1956 by the French demographer Sully Ledermann.^13^ Ledermann argued that the level of alcohol consumption within a population was related to the extent of alcohol problems within that population. As the total amount of alcohol consumed increased, so too did the number of individuals with alcohol problems. Reducing the amount of alcohol consumed by everyone, whether a problem drinker or not, would result in better health outcomes overall. This thesis, as we shall see, was controversial, but the population level approach to public health problems was endorsed and further developed by leading epidemiologists, like Geoffrey Rose.^14^ Moreover, a population view of alcohol ‘disabilities’, one that stressed the environment as well as individual responsibility, found support at the global level, especially at the World Health Organisation.^15^

A central reason why the Ledermann thesis was unpopular within sections of the UK government was because it implied that in order to reduce consumption, alcohol should become harder and/or more expensive to obtain. This approach seemed to conflict with the interests of a third public: citizen-consumers. A relationship between citizenship and consumption had existed since at least the nineteenth century, but in the second half of the twentieth century citizen and consumer identities moved even closer together.^16^ Consumerist principles such as the ability to complain and the right to information found their way into public services like housing and health care. Facilitating the power of citizen-consumer’s to make use of high quality goods and services became paramount. Viewed in this light, any restrictions on a drinker’s ability to consume alcohol were not only a limitation to traditional ideas about individual liberty, but also placed unacceptable restraints on consumption. Making alcohol more expensive or increasing restrictions on its sale was antithetical to a trend that encouraged the provision of high quality inexpensive goods and services. Of course, a key role for the state was to regulate consumption and make sure that products could be consumed safely, and alcohol undoubtedly posed dangers to individual and collective health. But instead of increasing restrictions, a more acceptable way to curb alcohol use was to appeal to the rationality of the citizen-consumer, to provide information and education in order to allow him or her to consume alcohol sensibly. Health education offered a way to alert the public to the risks posed by alcohol without restricting the choices of citizen-consumers.

## Health Education: A Brief History

Attempts to educate the public about dangers to health and ways to ameliorate these had long been part of public health.^17^ Health education, however, assumed new importance in the wake of the bacteriological revolution at the end of the nineteenth century, as the behaviour of individuals, as well as the environment, became crucial to understandings of how disease spread.^18^ As a result, in the early twentieth century, public health policy makers and practitioners in Britain made use of health education to attempt to inculcate personal hygiene and preventive habits amongst the population. Such efforts were also aimed at promoting morality and good citizenship.^19^ Health was thus an individual responsibility and a public duty.^20^ At the local level, Medical Officers of Health carried out health education work including lectures, exhibitions, health weeks and the creation of visual material such as posters and leaflets.^21^ At the national level, the Central Council for Health Education (CCHE) was established in 1927 to attempt to coordinate the field. The CCHE was funded by subscriptions from local authorities, not central government, and the Council lacked leadership for much of its life.^22^

By the mid-twentieth century, however, there were signs of a change of direction for health education. In part, this was a response to shifting patterns of morbidity and mortality. As infectious disease in the West declined, chronic conditions, often linked to individual behaviour, appeared to increase.^23^ Behaviour was regarded as a cause of disease, not just a way of spreading it. This warranted a new approach within health education. One of the first areas where such a move can be observed is around smoking and cancer.^24^ In the early 1950s, the work of Austin Bradford Hill and Richard Doll established a causal link between smoking and lung cancer. The obvious way to reduce the incidence of lung cancer was to encourage individuals to stop smoking. Although some health educators saw the public as irrational and fearful, especially with respect to cancer, others saw the public as reasonable and educable.^25^ In the 1950s, health education messages around smoking tended to favour the latter approach, and appealed to the smoker as a rational individual, presenting him (at this point messages were directed largely at men) with information about the potential dangers of smoking, but leaving it up to him to decide what to do.^26^

By the 1960s, a further shift was detectable as the list of behaviours that could cause ill health began to grow. In 1964, the Central and Scottish Health Services Councils published a report on health education. Named after its chairman, Lord Cohen, the Cohen Report argued that ‘Health education must do more than provide information. It must also seek to influence people to act on that advice and information given.’^27^ The report recommended moving away from what it termed ‘specific action campaigns’, such as educating the public about vaccination, and towards areas of ‘self-discipline’, such as smoking, overeating and exercise. The Cohen report also suggested a change of tactics, recommending that more use be made of the mass media, and called for the creation of ‘a strong central board’ to oversee health education. The government accepted the Cohen report’s recommendations, and in 1968 the Health Education Council was established. The HEC took over the CCHE’s functions, but instead of being funded by local authorities, financial support came from central government, although technically the Council was independent of its supporting department, the DHSS.^28^ The HEC decided that its ‘first concern should be with the prevention of common diseases which impair working capacity, cause distressing disability and premature death’.^29^ This included conditions related to behaviours like smoking and alcohol consumption.

Individual behaviour also figured centrally in a series of major reports on the state of public health and what to do about it. In 1976, a DHSS booklet entitled, *Prevention and Health: Everybody’s Business*, asserted that ‘We as a society are becoming increasingly aware of how much depends on the attitude and actions of the individual about his health. Prevention today is everybody’s business.’^30^ Emphasis was placed on preventing the development of disease in order to eliminate unnecessary suffering and reduce the financial burden of ill health. Health Minister David Ennals told the Royal Society of Health that ‘The types of change that are required in individual behaviour and habits in relation, for example, to smoking, drinking, eating and driving, cannot be brought about by Government action alone. To achieve significant and lasting changes in attitudes and life-style we must look increasingly to health education.’^31^ Health education was to have a central place in the new, preventive approach. The government’s 1977 report, *Prevention and Health*, stated that ‘Health education is one of the most important aspects of preventive medicine. It can contribute significantly to the public’s understanding of ill-health and its prevention and of the value of adopting healthy living habits.’^32^

Although individually focused health education designed to encourage personal prevention was the dominant method for dealing with public health problems in 1970s Britain, there were signs of an alternative approach in the making. The social, economic and environmental determinants of health began to attract increased attention, especially at the global level through the World Health Organisation.^33^ By the 1980s, the notion of ‘health promotion’ began to replace that of ‘health education’.^34^ Health promotion was about developing ‘positive health’—health as more than the absence of disease—and preventing illness rather than simply treating it. More traditional health education tactics, such as informing people about particular conditions and ways to prevent them, could be part of health promotion, but health promotion also encompassed a range of other techniques including working with communities to develop healthy environments.^35^ In the UK, such an approach manifested itself in various ways. There were attempts to develop a specialist training programme for health promoters and also efforts to underscore the relationship between poverty and ill health.^36^

Some saw the appearance of health promotion as part of a ‘new public health’ that emphasised both individual behaviour and structural factors as the leading causes of ill health. John Ashton (later President of the Faculty of Public Health) and health promoter Howard Seymour argued that ‘the New Public Health is an approach which brings together environmental change and personal preventive measures . … Many contemporary health problems are therefore seen as being social rather than solely individual problems.’^37^ Yet, the meaning of the new public health was (and continues to be) a ‘moving target’.^38^ When the ‘new public health’ came into being and precisely what was ‘new’ about it has prompted much debate. Dorothy Porter contends that the origins of the new public health can be traced to the late 1950s, rooted in social medicine and especially the work of Jerry Morris.^39^ In contrast, Niyi Awofeso suggests that the era of the new public health did not really begin until the 1990s.^40^ Other critics have taken issue with the nature of the new public health itself. Structural approaches did not always sit easily alongside efforts aimed at getting people to change their behaviour. Sociologists such as Alan Petersen, Deborah Lupton and David Armstrong saw the new public health as a way of disciplining individuals, of increasing surveillance and blaming victims for their plight.^41^ Tensions between and within these meanings of the new public health can be observed in differing approaches to health education, and especially in efforts to deal with alcohol as a public health problem.

## Alcohol: a Public Health Problem?

The consumption of alcoholic beverages and their affects on drinkers was not a new area of governmental concern in the 1970s. Alcohol had posed problems in terms of public order, danger to health and morality for centuries. During the nineteenth century, the habitual consumption of alcohol came to be seen as the disease of ‘alcoholism’, comprising both medical and moral elements.^42^ There were public health dimensions to the alcohol issue, especially around the impact drinking had on industrial production, but drink was not seen as a public health problem. The temperance movement, for instance, intersected rarely with those pressing for sanitary reform.^43^ Alcohol consumption and alcohol problems attracted relatively little attention in the early decades of the twentieth century. It was not until the 1950s, when there was an apparent rise in the number of alcoholics, that the disease-based view of alcoholism was ‘re-discovered’, prompting the establishment of dedicated treatment units for individuals with alcohol problems.^44^

A wider appreciation of the difficulties that alcohol could cause began to emerge in the 1960s. Initially, the focus was on drink driving. Measures such as the introduction of the breathalyser in 1967 were designed to protect the public from intoxicated drivers and reduce the number of motor vehicle accidents.^45^ Towards the end of the decade, a more distinct public health view of alcohol problems started to appear. This was prompted by a marked growth in alcohol consumption during the 1960s and 1970s, and with it an increase in alcohol-related illnesses such as cirrhosis of the liver.^46^ Alcohol consumption almost doubled between 1950 and the mid-1970s, rising from 5.2 litres of pure alcohol per person per year to 9.3 litres.^47^ Deaths from liver cirrhosis increased from just over 20 per million in 1950 to more than 40 per million by 1970.^48^ Alcohol clearly posed a danger to public health, but it was not the established authorities and institutions within public health policy making and practice that pushed alcohol on to the public health agenda. Instead, a distinct ‘alcohol policy network’, made up of doctors and researchers who specialised in alcohol and addictions, voluntary organisations and sympathetic civil servants, were instrumental in getting the government to take alcohol issues seriously.^49^ This alcohol policy network was able to take the lead in defining alcohol as a public health issue because the traditional bastions of public health practice and policy making were in disarray in this period. The key public health official, the Medical Officer of Health (MOH), had undergone a gradual diminution in status following the establishment of the NHS.^50^ The position of MOH was scrapped altogether when public health services moved out of local government following the reorganisation of the health service in 1973, although it was later replaced with the Director of Public Health role when public health ‘returned’ to local government in 2012.^51^ Academic public health was also experiencing significant change, most notably around the uses of epidemiology to demonstrate causal links between behaviour and disease.^52^

Indeed, it was an epidemiological view of alcohol consumption that helped redefine alcohol as a public health issue. Key members of the British alcohol policy network championed the Ledermann thesis and asserted that the extent of alcohol problems was related to the total level of alcohol consumption within the population. This epidemiological approach to alcohol prompted a series of government reports and investigations by medical professional bodies throughout the late 1970s and early 1980s. As will be discussed in greater detail below, there was some support for the idea that tax should be used to increase the price of alcohol (or at least not let it decline further in real terms) so as to decrease population-level consumption, and therefore alcohol related harms. Such an approach was controversial: a report produced by a government think tank that had suggested taxation be used to control the price of drink was suppressed.^53^ The government was reluctant to use tax policy in this way, and were fearful of the economic impact such measures would have on the drinks industry, tax revenue and jobs.

Nonetheless, something needed to be done about alcohol problems. The apparent solution was to focus on health education. Here was something that all parties, including health professionals, government and the alcohol industry, could agree on. Alcohol health education was, according to Rob Baggott, an ‘island of consensus’.^54^ Yet, this ‘island of consensus’ was really a mirage. A close examination of the development of alcohol education in the 1970s demonstrates that there was little unity about the tactics to be used or their effectiveness. This casts doubt not only over alcohol education, but also over health education in general and the nature of the relationship between public health and the public.

## The HEC’s North East Campaigns on Alcohol Education, 1974–1981

In the early 1970s, the newly established HEC decided to mount a health education campaign on alcohol. Such a move can be explained by the growing concern about alcohol problems within government, but was also rooted in the HEC’s view of public health and its role in promoting it. The HEC saw health as ‘more than bodily fitness—that ultimately our concern was to help people live in a state of harmony with themselves and with the community as a whole’.^55^ Alcohol problems fitted within this approach. In November 1973, the HEC agreed to run a pilot anti-alcohol campaign in the north east of England.^56^ The Council was tasked with delivering health education nationally and locally, although most of their work at the local level was restricted to providing information, leaflets and guidance to local authorities.^57^ The north east campaigns on alcohol were somewhat different: they were intended to test the approach before rolling the programme out to other regions.

Why the north east region was chosen for the pilot is unclear. The fact that the area had the highest alcohol consumption levels for men in the UK was later used to justify selection, although this irritated local service workers who felt that problems in the north east were no worse than anywhere else in the country.^58^ The selected region was also coterminous with the boundaries of the Area Health Authority and the Tyne Tees television area, something that made the distribution of TV advertisements easier. The HEC’s alcohol education programme in the north east was divided into three distinct phases. The first was in 1974; the second between 1977 and 1979; and the final phase occurred in 1981. Each campaign adopted a different approach, and the difficulties encountered reveal varied aspects of the problems underpinning alcohol health education.

### 

The first stage of the HEC’s anti-alcohol programme began in October 1974. It aimed to: first, increase professional awareness of alcohol problems; and secondly, to establish the feasibility of health education about alcohol problems.^59^ The campaign cost £88,000, with £60,000 being spent on TV, press and poster advertisements.^60^ The campaign material was designed by the London-based advertising agency, Saatchi and Saatchi. The agency was one of the first to fully appreciate the value of TV advertisements for reaching a large audience and was well known for their imaginative approach.^61^ The HEC had used Saatchi and Saatchi previously to create anti-smoking material, including a controversial image of a naked, pregnant woman smoking.^62^

**Fig. 1:**
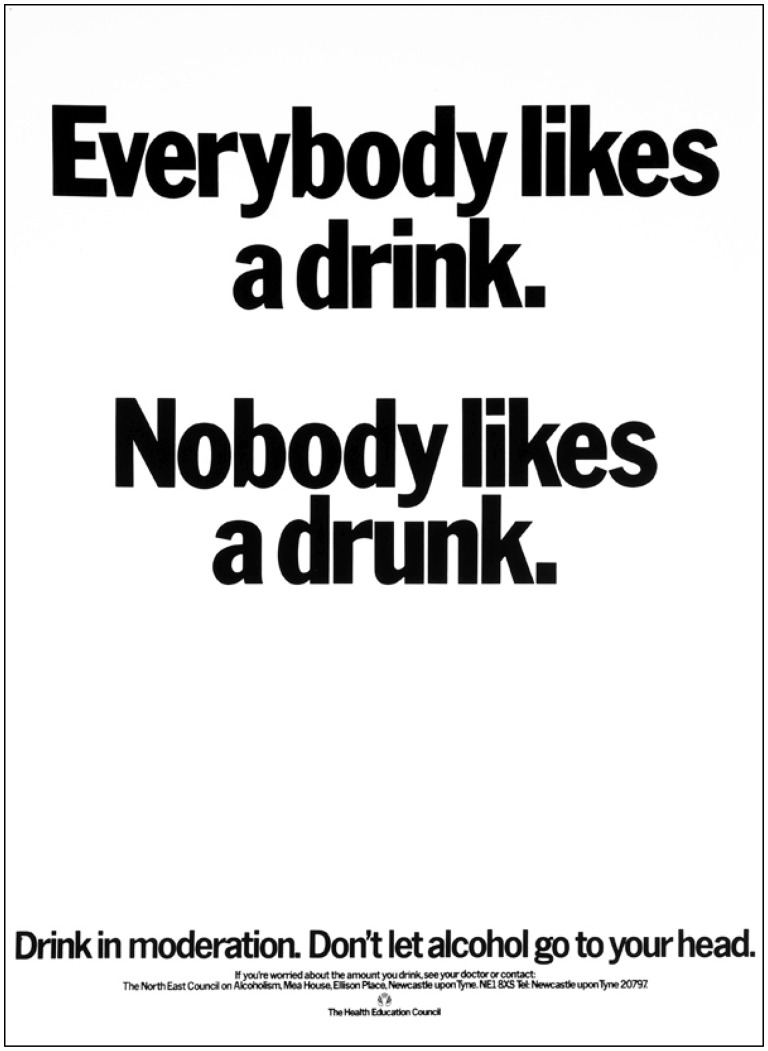
‘Everybody likes a drink. Nobody likes a drunk’. Saatchi & Saatchi for the Health Education Council, 1974.

The advertisements that the agency designed for the anti-alcohol campaign were no less provocative. Based around the tagline ‘Everybody likes a drink. Nobody likes a drunk.’ the advertisements attempted to convey some of the dangers of heavy drinking; the signs and symptoms that were indicative of problems due to heavy drinking; and where to get help (Figure 1).^63^ The posters used for the campaign were stark and simple, with no visual imagery beyond the slogan itself, and a further exhortation to ‘Drink in moderation’ and ‘Not let alcohol go to your head.’ The HEC felt that the central slogan ‘would be a powerful and positive message to adopt, without exposing the Council to accusations of being killjoys’.^64^ Yet, not everyone agreed. Local psychiatrist Anthony Thorley argued that the slogan was ‘criticised and misunderstood by many North-easterners. Not everybody does like a drink. People are not all agreed as to what a “drunk” is. One man’s “sensible drinking” is another man’s stupidity.’^65^ The Medical Council on Alcoholism and the Alcohol Education Centre also objected to the tag line, and would have preferred that it read ‘Almost everybody likes a drink’.^66^

Criticism of the campaign went beyond its tagline. The campaign was intended to be a piece of primary prevention—that is, it was designed to stop alcohol problems from developing. Yet, the focus of the advertisements, and even the way that the agency and the HEC described the campaign, suggested that the target group was those already experiencing alcohol problems, such as alcoholics and heavy drinkers, rather than the general population. The HEC tended to refer to their efforts as the ‘anti-alcoholism campaign’, and saw the fact that over 900 people contacted treatment services in the wake of the campaign as a sign of its success.^67^ On the ground in the north east, local alcohol agency workers were less convinced. Services were overwhelmed and they lacked the capacity to assist everyone who came forward for help.^68^ An evaluation of the campaign suggested that whilst penetration was high—most people interviewed recalled seeing the advertisements on TV or in the newspapers—there was little lasting change in attitudes towards drinking or drinking behaviour.^69^

### 

**Fig. 2:**
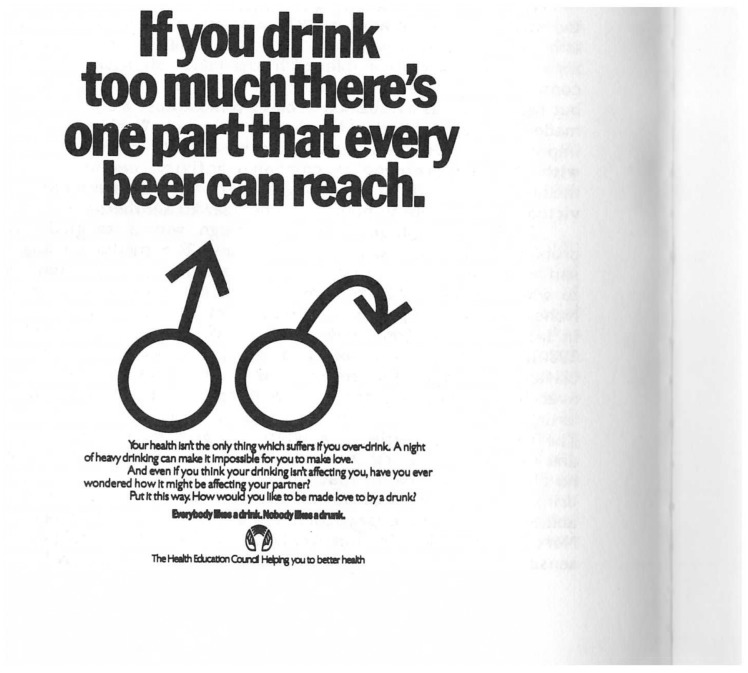
‘If you drink too much there’s one part that every beer can reach.’ Saatchi and Saatchi for the Health Education Council, 1979.

The HEC took on board some of the criticisms made of the 1974 campaign when designing a second phase, which ran between 1977 and 1979. This stage of the campaign had similar aims as before, and initially utilised the same material, but later developed new resources under the slogan ‘It’s always the boozer who’s the loser.’ Fresh visual and audio-visual material was commissioned by the HEC, who again made use of Saatchi and Saatchi. The agency produced ‘playlets’ which were shown on Tyne Tees TV and in local cinemas. These advertisements were criticised by local agencies, which regarded them as still too focused on alcoholics rather than on everyday drinkers. Moreover, the campaign betrayed a lack of understanding of the local population. Voices of the actors in the advertisements had Yorkshire accents rather than those of people from the north east, and the content of the commercials was too geared to a ‘middle class view of life’.^70^ Thorley argued that one of the posters, which featured a picture of a manicured female hand reaching for a bottle of vodka, was a ‘jet-set’ image that did not resonate in the north east. Another poster focused on the effect that alcohol could have on men’s sexual performance (Figure 2). Making use of the universal symbol for male, the poster suggested that having too much to drink could result in erectile dysfunction, or ‘brewer’s droop’. The poster won an advertising prize, but not everyone viewing the poster understood what the symbols meant.^71^ Thorley suggested that ‘in the North-east the vast majority of people had no idea at all what the symbols represented. One wit even queried whether it represented a crashed Volvo car!^’^^72^

**Fig. 3:**
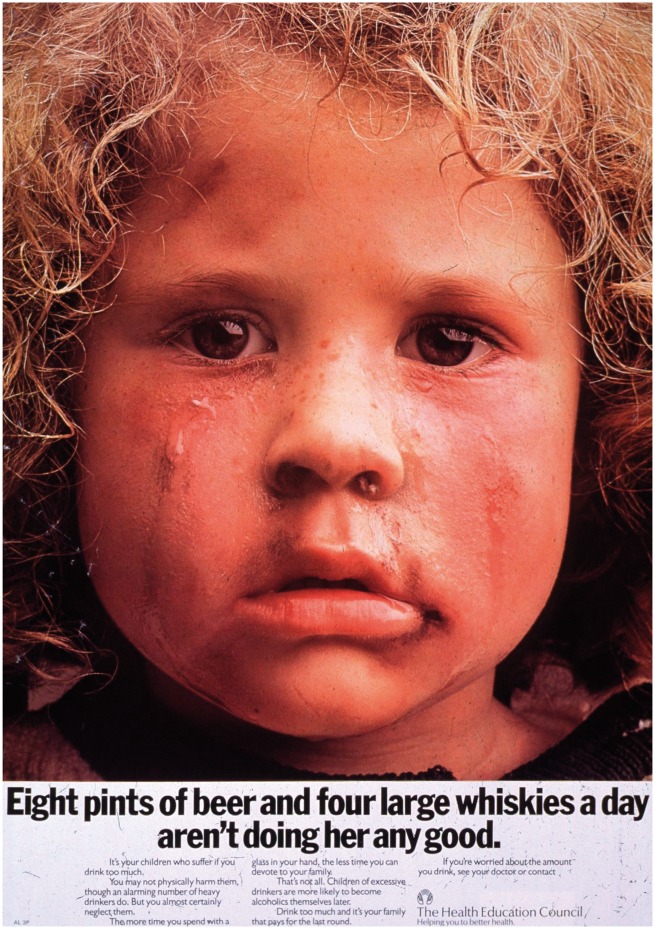
‘Eight pints of beer and four double whiskies a day aren’t doing her any good.’ Saatchi & Saatchi for the Health Education Council, 1981.

Another poster featured an image of a crying child (Figure 3). Her dirty, bruised face was streaked with tears, and the strapline read: ‘Eight pints of beer and four large whiskies a day aren’t doing her any good.’ Once again, Thorley felt that the image was misunderstood, and though the poster ‘became well known throughout the region’ a ‘minority thought it was the *girl* who had been drinking!’^73^

Misunderstood or not, these posters indicated a change of tactics and focus. Both posters attempted to appeal to the emotions of the viewer in order to provoke reflection on the amount of alcohol s/he consumed. The ‘brewer’s droop’ poster made use of humour to encourage the viewer to think about the consequences of heavy drinking for themselves and for their sexual partner. The ‘battered child’ poster focused on the damage alcohol could cause to an ‘innocent victim’, a trope found in nineteenth-century temperance material and also within more contemporary public health campaigns, such as those around smoking.^74^ The posters drew attention to the wider consequences of alcohol consumption beyond those impacting upon the individual drinker themselves, thus emphasising the social dimension to the alcohol problem, rather than purely the medical one. This was reinforced by the impression that the posters appeared to be aimed at ordinary (albeit ‘heavy’ or ‘excessive’) drinkers rather than alcoholics.

The second phase of the campaign came to an end in 1979. According to an evaluation of the campaign, the HEC said that they decided to abandon their efforts due to lack of action and coordination on the ground, something denied by those in the north east.^75^ Thorley contended that ‘By 1979 it was clear that the media work, now costing almost half a million pounds, was ineffective and increasingly embarrassing to all concerned.’^76^ For their part, Saatchi and Saatchi were also dissatisfied with the campaign, as they found the central brief, to focus on encouraging moderation in drinking, was a difficult task to fulfil.^77^ Indeed, the campaign material gave little indication as to what ‘moderate’ drinking consisted of. The ‘battered child’ poster did appear to suggest that eight pints of beer and four large whiskies a day was ‘too much’, but the setting of limits to alcohol consumption was to form a more central part of the campaign’s third phase, in 1981.

### 

**Fig. 4:**
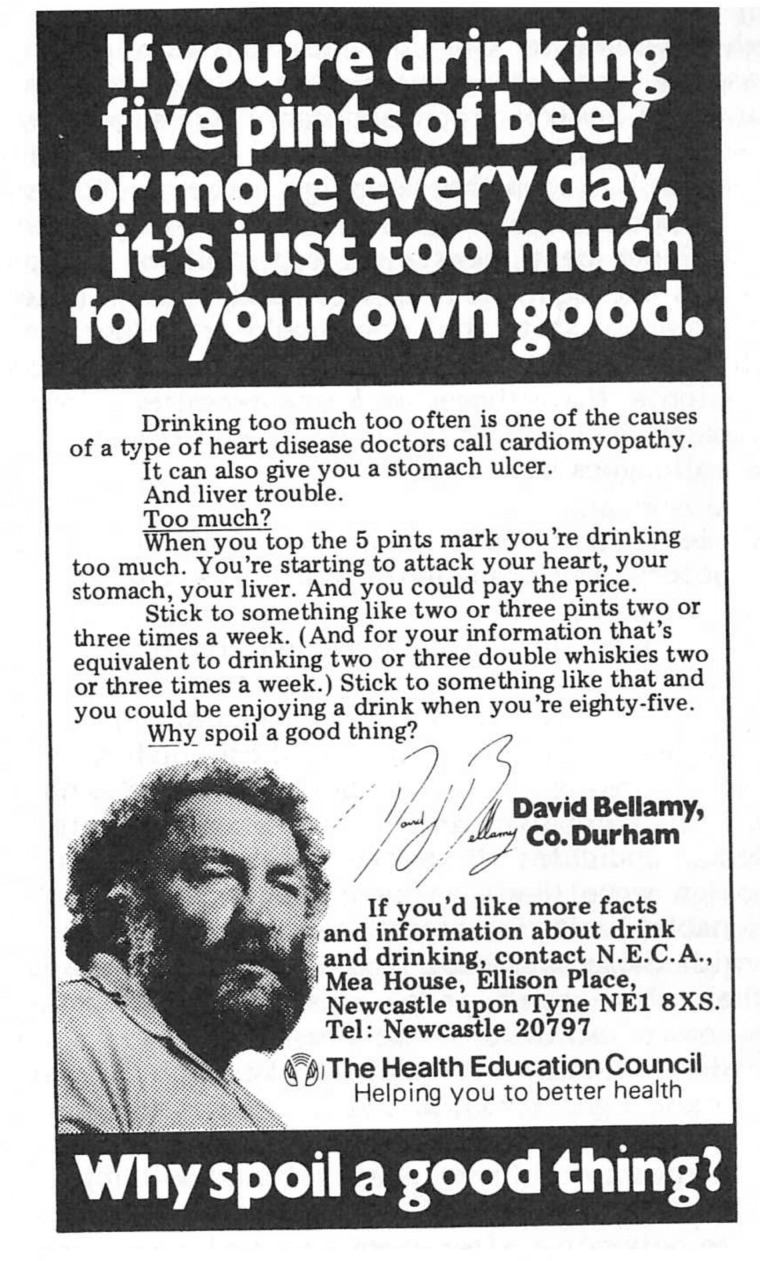
‘If you’re drinking five pints of beer or more everyday … ’ Redlands for the Health Education Council, 1981

The final stage of the HEC’s anti-alcohol campaign in the north east was framed around a desire to promote ‘moderate drinking’. Those involved in devising the campaign wanted it to focus on heightening awareness of alcohol problems rather than cutting the consumption of alcohol per se.^78^ The HEC dropped Saatchi and Saatchi, and instead made use of a Newcastle-based advertising agency, Redlands. The agency devised new campaign materials featuring local TV presenter and botanist, David Bellamy. Bellamy was chosen by Redlands because they felt that he would be seen by the public as intelligent and honest, but also able to connect with the intended audience as he was from the north east and a drinker himself.^79^ The advertisements offered guidance on how much alcohol was ‘too much’ (five pints of beer or more) and also suggested a level of moderate consumption as being ‘something like two or three pints two or three times a week’ (Figure 4). Indeed, the benefits of moderate alcohol consumption were tacitly acknowledged by the campaign’s tagline ‘Why spoil a good thing?’.

Some saw the campaign’s issuing of clear guidance on levels of alcohol consumption as more informative and less moralising than previous messages.^80^ The setting of drinking limits was, however, controversial. There was little agreement amongst experts about what a ‘safe’ level of drinking consisted of. In their 1979 report, the Royal College of Psychiatrists suggested that four pints, four double whiskies or one bottle of wine a day ‘constitute reasonable guidelines of the upper limit of drinking’.^81^ Yet, other experts were concerned that setting an upper limit would encourage people to drink up to that level in the belief that their behaviour could do no harm.^82^ Devising guidance around safe alcohol consumption limits became a feature of alcohol policy in the mid-1980s, but this campaign was one of the first to attempt to communicate information about ‘sensible drinking’ to the wider public.

A survey conducted in the north east in the wake of the ‘Bellamy campaign’ suggested that the core message around moderate drinking did get through to the local population. More than two-thirds of those interviewed recalled the campaign, and all but four of the 750 respondents were able to remember something relevant when questioned about the main message of the campaign. When asked if the campaign had changed their behaviour, 12.7 per cent claimed that it had, but only three people said that they had actually tried to drink less.^83^ As an evaluation of the campaign pointed out, it had not been designed to change behaviour, and based on its original goal of raising public awareness about moderate alcohol consumption, the campaign could be judged a modest success.^84^ However, the HEC’s paymasters, the DHSS, were less convinced. The department and its ministers were aware that changing behaviour was challenging and time consuming. In 1981, the Secretary of State for Social Services, Patrick Jenkin, told a meeting of the National Council on Alcoholism that ‘It is difficult to modify social attitudes and difficult to measure what, if anything, has been achieved. Health education is a long haul.’ But he also remarked that ‘At a time when money is clearly limited, Ministers and all concerned need to be convinced that the available resources will be used to good effect.’^85^ The HEC were under pressure to demonstrate the cost-effectiveness of their work, but they were unsure that alcohol health education would result in reduced alcohol consumption, at least directly. The Council’s alcohol programme strategy for 1982–83 argued that many forces influenced alcohol consumption, and as a result ‘health education *by itself* [original emphasis] has only a limited ability to reduce it’. Other measures, such as greater control of alcohol, and aiming to reduce per capita consumption, also had a part to play in dealing with alcohol related harm.^86^

Taken together, the three phases of the HEC’s alcohol education campaign in the north east points to an evolution in targets, techniques and tactics. In the first phase of the campaign, the target group seemed to be alcoholics, or the ‘drunk’. In the second phase, the target group was the ‘boozer’ or the ‘heavy drinker’. In the final phase, it appeared that a wider drinking public was the target, with the desire to promote ‘sensible’ or ‘moderate’ drinking. The techniques also altered over time, with humour and emotional entreaties giving way to a more ‘rational’ approach, appealing to the drinker as a ‘sensible’ individual able to moderate their behaviour. Such changing techniques spoke also to changing tactics, with a more specific sense of the kinds of behaviour that should be encouraged or discouraged emerging by the end of the period. These shifts reflected broader developments at the policy level that will be explored in the remainder of the article, but at the same time there was also a lack of confidence about health education itself. Significant doubts were expressed, not least by the HEC, about the ability of health education to shrink alcohol consumption. Other means, such as reducing drinking at the population level, seemed to offer an alternative solution.

## The Report of Advisory Committee on Alcoholism on *Prevention*, 1975–1977

The best way to prevent the development of alcohol problems, including the role of population level measures and health education, was examined by a number of expert committees in the 1970s. A key report was produced by the Advisory Committee on Alcoholism (ACA), which was established in 1975 to advise the government on the provision of services relating to alcoholism. According to Betsy Thom, their terms of reference were vague, and the committee were able to interpret their brief quite widely, examining not only treatment services, but also the prevention of alcohol problems.^87^ As a result, the ACA were interested not only in alcoholics and heavy drinkers, but also those who might develop drinking problems, and the consumption of alcohol within the population more broadly. In their report the ACA argued that ‘we have to consider not only the affected individual, those who come into contact with him, and vulnerable groups, but also deep rooted attitudes, assumptions and traditions which blind people to the wide range of problems caused by alcohol misuse’.^88^

The ACA’s expansive interpretation of the potential damage that alcohol could cause led it towards a broad understanding of the ways in which such problems could be prevented. The committee’s decision to focus on prevention was, however, ‘against the Chairman’s wishes and our [the DHSS’s] advice’. The DHSS were well aware that the ACA was likely to stray into areas that were the concern of other government departments, such as MAFF, which they saw as ‘the sponsoring department for the drinks industry’.^89^ A particular flash point was the Ledermann thesis and the notion that introducing measures to decrease alcohol consumption throughout the population could reduce drink problems. At the ACA’s first meeting they accepted Ledermann’s arguments, stating that ‘the available facts pointed strongly towards the need for a reduction in per capita consumption of alcohol as one of the objectives of any preventive strategy’. But, at the same time, the committee were also aware of the potential political and social consequences of such an approach. They noted that ‘Increasing the price of alcohol in real terms to a point where consumption was substantially affected would be difficult politically and might cause secondary poverty.’^90^ As a result, the committee did not suggest any changes to fiscal controls or the licensing laws; instead they recommended that ‘alcohol should not be allowed to become cheaper in real terms’.^91^

Alongside this moderate form of price control, the committee proposed that more effort be put into health education. In their final report the ACA recommended that ‘Health education designed to alert people to the dangers of alcohol and to discourage excessive drinking should be encouraged and expanded.’^92^ In the discussions leading up to the publication of the report, however, health education had occupied a more controversial position. The psychiatrist and addiction researcher Griffith Edwards ‘had considerable reservations about any campaign which attempted to change people’s behaviour’. Edwards was in favour of the introduction of greater controls on the price and availability of alcohol, and he suggested that ‘any campaign which was mounted should attempt to educate the public about the need for controls over the availability of alcohol as a means of preventing alcoholism’.^93^ Not everyone on the committee agreed, and at a later meeting (where Edwards was absent) they began to move towards an approach that emphasised ‘safe’ or ‘healthy’ drinking.^94^ The committee expressed some doubt about the ‘value of referring to “healthy drinking” or “safe drinking levels”’ as ‘the message conveyed was so complex that it seemed likely to be misunderstood’.^95^ Nonetheless, the ACA did touch on the issue in their report, suggesting that there was a need to ‘define a level of heavy drinking and to discourage drinking above that level’. They even made a tentative suggestion as to what this level should consist of, noting that a daily intake of 15cl of ethanol, equivalent to about half a bottle of spirits or 8–10 pints, was ‘generally regarded as unsafe’.^96^

The ACA’s provisional approach, and reluctance to either offer firm guidelines on ‘safe drinking’, or wholly endorse stronger population level control measures, was a result of their recognition that such issues were ‘controversial’ and ‘sensitive’. The Committee were unsure about the extent to which it was ‘justifiable to interfere with the activities of drinkers on account of those who may cause or come to harm’. The issue was not ‘thought to be one on which a Government could impose its will without paying the most careful regard to the views of the people’.^97^ As a result, the ACA argued that ‘stricter controls cannot and should not be introduced without informed public discussion’. Moreover, ‘The problems resulting from alcohol misuse have not yet been widely enough discussed: we believe that the public should be given more information, including an estimate of the true cost of alcohol misuse to society so that it can reach a realistic view of the restraints that should be placed on drinking.’^98^ This approach was also endorsed by the DHSS’s booklet, *Prevention and Health: Everybody’s Business*, which stated that ‘The best combination of strategies for our society, and the attitudes to alcohol which should be encouraged in it, are matters which deserve public discussion.’^99^

Indeed, some level of public debate about alcohol health education campaigns was already taking place. Most of the broadsheet newspapers simply summarised the key findings of the ACA’s report, but some of the more libertarian publications offered editorials on the wider issue of health education. An article by Colin Welch in the *Daily Telegraph* was highly critical of government-backed health education efforts against smoking and drinking, which he saw as a ‘sinister step towards tyranny’. Taylor asserted that ‘When the British people imposed on the State the duty of caring for all our ailments free of change we forgot that wise adage—*there is no free lunch*. … For the State at that very moment acquired the right to order us to live healthy lives—to eschew this or that substance or practice’.^100^ ‘Peter Simple’, also writing in the *Daily Telegraph*, took a similar tack. He stated that government plans to put a health warning on the labels of alcoholic drinks was an ‘idiotic message’ and a ‘symbol of bureaucratic welfarism^’^^.^^101^ Others in the media, however, were less critical of such an approach. Reporting on a speech made by Ennals, where the minister had asked whether or not alcohol problems should be tackled more ‘vigorously’, *The Economist* responded ‘The answer surely is yes: and for a start his [Ennals’] advisory committee on alcoholism has suggested preventive measures that would not conflict with the enjoyment of normal drinking.^’^^102^ It is impossible to know the extent to which the wider public shared the views expressed by ‘Simple’ and Taylor, but their presence did suggest that there was some level of feeling that introducing stronger control measures on alcohol might be an unacceptable restriction of liberty, something the ACA itself had acknowledged. There was a perceived need for public debate about the approach to be taken to alcohol, and the extent to which individual drinking should be curbed for the public good.

## *Drinking Sensibly*, 1977–1981

An opportunity for dialogue about the response to alcohol was provided by a ‘nationwide debate’ initiated at the end of 1977. When launching the debate, Ennals said that there were questions about alcohol that ‘we must all ask ourselves’. Was it the role of Government, he wondered, ‘to concern itself with personal behaviour—or do you believe the Government has a duty to represent the interests of the community and seek to contain a growing ill?’ Should Government, Ennals inquired, ‘impose a much bigger tax on all intoxicating drink as a deterrent to drinking, or would this be unfair to the majority who are sensible drinkers’?^103^ A ‘consultative document’, to be prepared by the DHSS, was intended to ‘outline for discussion the arguments for and against various possible preventive measures’ and it was hoped that ‘the ensuing debate will assist the Government to draw up firm proposals for improvement^’^^.^^104^ Work began on the document in 1977, and it was intended that the text be published in 1978, but it took until 1981 for the final report, *Drinking Sensibly*, to appear.

The long gestation of *Drinking Sensibly* was the result of significant interdepartmental tension. The central difficulty surrounded the control of alcohol prices, the impact that this would have on consumption, and whether or not taxation should be used to increase the price of drink. Not everyone within the DHSS was convinced of the Ledermann thesis, but by 1976 key officials and the Minister were of the opinion that ‘there is sufficient evidence available to link price, consumption and damage as to make it desirable that drink in all its forms should not become *cheaper*^’^^.^^105^ Such views found their way into an early draft of the consultation document. The document stated that ‘There seems little doubt that lowering the price of alcoholic drink does tend to encourage greater consumption, while raising prices leads to a fall-off in the amount people drink.’ The draft was equivocal on whether or not tax should be used to increase price—this was something for the government and the wider public to decide—but the document implied that inexpensive alcohol meant that the problem would worsen: ‘Cheaper and cheaper drink prices would severely hamper efforts through health education and other means to tackle the problem of alcohol misuse and perhaps make all such efforts abortive.^’^^106^

Other government departments did not see things the same way. The Department of Trade, Customs and Excise, MAFF, the Home Office and the Treasury all had difficulties with aspects of the draft text on alcohol taxation and price disincentives. As a letter from an official at Customs and Excise noted, ‘It is clear that this chapter [on tax and price control] raises issues about which there is considerable disagreement between Departments.^’^^107^ The Department of Trade were concerned about ‘the practicability and desirability of seeking to hold down the consumption of alcohol through action on prices, and the implications for competition and consumer choice of any more restrictive approach to licensing^’^^.^^108^ MAFF wanted the document to recognise the importance of the drinks industry to the economy. They wanted the document to ‘avoid suggesting that there is one single problem that can be dealt with by general solutions. General solutions would penalise unfairly the majority of sensible drinkers and without any guarantee that the number of problem drinkers would be reduced.^’^^109^ The Treasury sought to delay release of the document, and possibly prevent it from being published at all. The change of government in May 1979 offered an opportunity to ‘seek guidance from Ministers before a great deal of additional effort is put into revising the present draft’. Treasury officials noted that ‘The first question is whether the present Government will wish to publish any document along these lines; and we for our part would want to recommend to our Ministers that they should consider carefully the policy implications before coming to a firm decision.^’^^110^

The consultative document survived, but in a significantly modified form, and only after it was approved at Cabinet level.^111^ The section on price and tax was re-written substantially. No direct comment was made about the link between price and consumption; instead the final document simply summarised the recommendations made by other committees and reports, such as the ACA’s *Prevention*.^112^ Where the document was unequivocal, however, was on the issue of taxation. It stated that ‘Taking account of the economic as well as the health and social considerations, and bearing in mind the practical difficulties involved, the Government cannot accept recommendations that have been made for the systematic use of tax rates as a means of regulating consumption.^’^^113^ The possibility of using taxation to control the price of drink was not up for discussion. Indeed, the overall tone of *Drinking Sensibly* was not as ‘consultative’ or as open to ‘debate’ as intended originally. Although the text was billed as a ‘discussion document’ it was unclear how such discussion would take place. Instead, *Drinking Sensibly* was intended to ‘help clarify public views’ and offer ‘statements of the government’s position’.^114^

In any case, it does not seem as if *Drinking Sensibly* stimulated much public debate. The DHSS had intended that the document ‘be aimed at the intelligent layman, in the hope that the Press and TV will be sufficiently interested to follow up some of the points and so reach a wider audience.’^115^ Yet, they decided to publish the document with a plain cover, since the anticipated readership was ‘the influencers of opinion’ rather than ‘impulse buyers’.^116^ The media did report on the publication of *Drinking Sensibly*, but most of the newspapers just summarised the document’s key statements and highlighted the fact that the government was not recommending an increase in the tax on alcohol. *The Guardian* was alone in sounding a critical note: ‘The Government is to take no direct action—either by tax increases on alcohol or by curbing drinks advertising—to halt a dramatic rise in the misuse of alcohol.’ Instead, the ‘drive to curb abuse would rely entirely on voluntary effort’.^117^ The only other source of criticism came from the medical press. The psychiatrist and addiction expert Thomas Bewley, writing in the *British Medical Journal*, summed up his views on the document as ‘Drinking sensibly, perhaps. Thinking sensibly, no.^’^^118^

Although *Drinking Sensibly* provoked little public debate at the time, the report, and especially the notion of ‘drinking sensibly’ was important. The DHSS pondered long and hard over the title of the document. Alternatives included ‘Responsible Drinking’, ‘Sensible Drinking’, ‘Sensible Attitudes to Drinking’, ‘Preventing Alcohol Misuse’, and ‘Alcohol—The Right Balance’.^119^ Other suggested titles were less than serious, perhaps because they were developed in the run up to Christmas, 1979. It seems unlikely that ‘Not Only Mother’s Ruin’, ‘Down the Hatch or Down the Drain?’, ‘Don’t Trifle With Sherry’, ‘I Drink Therefore I Am’, ‘Steady as she Flows’ or ‘Blithe Spirit’ were ever in contention, but the debate over the title of the consultative document does draw attention to the way in which alcohol consumption was framed by the text.^120^ Although ‘Drinking Sensibly’ emerged as the victor, this was not defined in the final document. The text referred to ‘sensible attitudes towards the use of alcohol’ but it was not at all clear what these were.^121^*Drinking Sensibly* mentioned the Royal College of Psychiatrists’ suggestion that drinkers limit themselves to no more than four pints of beer, or four double spirits or one bottle of wine a day, but the report also pointed out ‘drawbacks’ to such an approach, such as the varied effect of alcohol on different people. The ‘sensible drinker’ may have been synonymous with the ‘responsible citizen’, as ‘Responsible citizens must consider in the light of these facts what they themselves can do to limit the harm to their own health and the health of others’.^122^ The rationality of the citizen-consumer was being appealed to, not only to protect his or her own health, but also that of the wider public.

## Conclusion

At the time of publication of *Drinking Sensibly* it seems as if the concepts of sensible drinking and the sensible drinker were still in development, but they came to hold significance in the later evolution of alcohol policy and alcohol health education. On a practical level, a more specific notion of what sensible drinking consisted of in terms of the amount of alcohol consumed began to develop in the latter half of the 1980s. Suggested daily limits had already been proposed by the Royal College of Psychiatrists, but in 1984 the HEC issued a pamphlet setting out the ‘safe limits’, to which people should restrict their drinking. ‘Safe limits’ for drinking were defined as 18 ‘standard drinks’ (equivalent to half a pint of beer, a small glass of wine or a single measure of spirits) a week for men and nine for women.^123^ In 1986 and 1987 the Royal College of Psychiatrists, the Royal College of Physicians and the Royal College of General Practitioners each published reports on alcohol, and all made the same recommendations with regards to consumption limits.^124^ The reports suggested that ‘sensible limits of drinking’ consisted of not more than 21 ‘units’ of alcohol a week for men, and not more than 14 units a week for women. A unit of alcohol was equal to 10ml or 8g of pure alcohol, or about half a pint of beer. In January 2016, the recommended weekly limit to alcohol consumption for men (previously 21 units) was brought into line with that of women (14 units).

Fluctuations in the recommended levels of alcohol consumption over time and the fact that many individuals continue to exceed these limits suggests that ‘sensible drinking’ was a mutable concept not a fixed category.^125^ Nonetheless, the unit, and with it sensible drinking, have survived as a cornerstone of alcohol policy for the last 30 years. Population level arguments about alcohol consumption have begun to reappear, but as the recent abandonment of the introduction of minimum unit pricing in England makes clear, such measures are bitterly contested.^126^ Public health policy and practice around alcohol continues to centre on health education, on persuading individuals to alter their drinking behaviour. Whether such measures ‘work’ is still open to question. There is evidence to indicate that health education can help push alcohol problems up the public and political agenda, but there is little to suggest that on its own health education can change drinking behaviour.^127^

In a sense, however, the debate about whether or not health education works misses a more fundamental point. The promotion of such a strategy was the result not only of the activities of vested interests, like the alcohol industry, but part of a more complex negotiation between different ‘publics’. Three publics can be found within the public health approach to dealing with alcohol: drinkers, the population and citizen-consumers. Drinkers were the targets of alcohol health education, but over the course of the 1970s, the type of drinker being aimed at changed. At the beginning of the decade, the focus was very much on alcoholics—the ‘drunk’ of the first phase of the HEC’s campaign in the north east. In the middle years of the 1970s, efforts centred on the ‘boozer’, a heavy drinker, but not necessarily an alcoholic. By the end of the decade, however, attention appeared to have shifted to the ‘sensible drinker’, to encouraging all drinkers to consume alcohol within proscribed limits. Such a move from focusing on the problem drinker to encompassing all drinkers brought a much larger group of people into the remit of alcohol policy. This shift was in line with a broader development within the ‘new public health’ to focus on the healthy as well as the sick, on disease prevention and on reducing the risk to health associated with certain kinds of behaviours. The ‘sensible drinker’ fitted within this approach.

The ‘sensible drinker’ was not, however, the only public at work and health education was not the only possible approach to dealing with the problems posed by alcohol. The Ledermann thesis introduced the idea that drinking levels within the entire population mattered and to reduce alcohol problems greater control of the price and availability of drink was required. The political and economic difficulties encountered by those who supported a population level approach were also bound up with the apparent needs of a third public: the citizen-consumer. Public health policy makers were concerned about implementing stronger levels of control over alcohol as this was thought to interfere with individual liberty, to be an unacceptable level of state intrusion into the private realm, but also an undesirable restriction on consumption. This could be seen as a re-run of an age-old dilemma for public health, about the extent to which it was acceptable to restrict individual freedom in order to ensure the public good, but it was also bound up with newer issues around consumer choice. Targeting individual behaviour through health education rather than addressing the wider environmental factors that encouraged people to drink could also be regarded as a repeat of earlier public health attempts to focus on the technical fix rather than address the social conditions that underpinned health problems. The ‘new public health’ was not, perhaps, so new after all.

But there was something different about the formulation of both the public and public health in post-war Britain. The case of alcohol health education suggests that there was more than one type of public, and more than one public health approach. The needs of each of these did not necessarily coincide, resulting in a confused response: one that targeted individuals at the same time as being concerned with the wider public, that wanted to get drinkers to consume alcohol ‘sensibly’, but was unclear about what this meant. Such confusion speaks to the limits of public health and the multi-faceted nature of the public. Not everybody likes a drink, but nobody likes to be told what to drink.

## Footnotes

1 George Cust, ‘Health Education about Alcohol in the Tyne Tees Area’, in J. S. Madden, Robin Walker and W. H. Kenyon (eds), *Aspects of Alcohol and Drug Dependence* (Kent: Pitman Medical, 1980), 117–22, 118.

2 J. Budd, P. Gray and R. McCron, *The Tyne Tees Alcohol Education Campaign: An Evaluation* (1983).

3 House of Commons, *First Report from the Expenditure Committee: Preventive Medicine, 1976/77* (London: HMSO, 1977), 35.

4 Advisory Committee on Alcoholism, *Report on Prevention* (London: HMSO, 1977).

5 Department of Health and Social Security (DHSS), *Prevention and Health. Drinking Sensibly* (London: HMSO, 1981).

6 Alan Petersen and Deborah Lupton, *The New Public Health: Health and Self in the Age of Risk* (London: Sage Publications, 1996); Clare Herrick, *Governing Health and Consumption: Sensible Citizens, Behaviour and the City* (Bristol: Policy Press, 2011).

7 Christopher Hamlin, ‘Public Health’, in *The Oxford Handbook of the History of Medicine*, ed. by Mark Jackson (Oxford: Oxford University Press, 2011), 411–28, 417; Jane E. Lewis, *What Price Community Medicine?: The Philosophy, Practice and Politics of Public Health Since 1919* (Brighton: Wheatsheaf Books, 1986), 5.

8 David Cantor, ‘Representing “the Public”: Medicine, Charity and the Public Sphere in Twentieth Century Britain’, in Steve Sturdy (ed.), *Medicine, Health and the Public Sphere in Britain: 1600–2000* (London: Routledge, 2002), 145–68, 160.

9 See <http://placingthepublic.lshtm.ac.uk>. accessed 1 June 2016.

10 A key text here was Kettil Bruun *et al.*, *Alcohol Control Policies in Public Health Perspective* (The Finnish Foundation for Alcohol Studies: New Brunswick, NJ: distributors, Rutgers University Center of Alcohol Studies, 1975).

11 J. N. Morris, *Uses of Epidemiology* (Edinburgh & London: E&S Livingstone Ltd., 1957), 16.

12 Nancy Krieger, ‘Who and What Is a “Population”? Historical Debates, Current Controversies, and Implications for Understanding “Population Health” and Rectifying Health Inequities’, *Milbank Quarterly*, 2012, 90, 634–81.

13 Betsy Thom, *Dealing with Drink: Alcohol and Social Policy in Contemporary England* (London; New York: Free Association Books, 1999), 109–11.

14 Geoffrey Rose, ‘Sick Individuals and Sick Populations’, *International Journal of Epidemiology*, 1985, 14, 32–8; Geoffrey Rose, *The Strategy of Preventive Medicine* (Oxford: Oxford University Press, 1992).

15 Robin Room, ‘The World Health Organization and Alcohol Control’, *British Journal of Addiction*, 1984, 79, 85–92.

16 See, for example, Frank Trentmann, ‘Citizenship and Consumption’, *Journal of Consumer Culture* 2007, 7, 147–58; Matthew Hilton and Martin Daunton, ‘Material Politics: An Introduction’ in Martin Daunton and Matthew Hilton (eds), *The Politics of Consumption: Material Culture and Citizenship in Europe and America* (Oxford: Berg, 2001), 1–32.

17 On pre-nineteenth century health education see George Rosen, *A History of Public Health*, expanded edition (Baltimore: Johns Hopkins University Press, 1993), 9, 34–36, 98–100.

18 Dorothy Porter, *Health, Civilization and the State: A History of Public Health from Ancient to Modern Times* (London: Routledge, 1999); David Armstrong, *Political Anatomy of the Body: Medical Knowledge in Britain in the Twentieth Century* (Cambridge: Cambridge University Press, 1983).

19 John Welshman, ‘“Bringing Beauty and Brightness to the Back Streets”: Health Education and Public Health in England and Wales, 1890–1940’, *Health Education Journal*, 1997, 56, 199–209.

20 Jane Seymour, ‘Not Rights but Reciprocal Responsibility: The Rhetoric of State Health Provision in Early Twentieth-Century Britain’, in *Assembling Health Rights in Global Context: Genealogies and Anthropologies*, ed. by Alex Mold and David Reubi (Abingdon; New York: Routledge, 2013), 23–41.

21 Martin Gorsky, ‘Local Leadership in Public Health: The Role of the Medical Officer of Health in Britain, 1872–1974’, *Journal of Epidemiology and Community Health*, 2007, 61, 468–72; John Welshman, ‘The Medical Officer of Health in England and Wales, 1900–1974: Watchdog or Lapdog?’, *Journal of Public Health Medicine*, 1997, 19, 443–50.

22 Max Blythe, ‘A History of the Central Council for Health Education, 1927–1968’ (unpublished DPhil, University of Oxford, Green College, 1987).

23 For a more critical account of this transition, see George Weisz, *Chronic Disease in the Twentieth Century: A History* (Baltimore: Johns Hopkins University Press, 2014); David Armstrong, ‘Chronic Illness: A Revisionist Account’, *Sociology of Health & Illness*, 2014, 36, 15–27.

24 Virginia Berridge, *Marketing Health: Smoking and the Discourse of Public Health in Britain, 1945–2000* (Oxford: Oxford University Press, 2007).

25 Elizabeth Toon, ‘“Cancer as the General Population Knows It”: Knowledge, Fear, and Lay Education in 1950s Britain’, *Bulletin of the History of Medicine*, 2007, 81.

26 Virginia Berridge and Kelly Loughlin, ‘Smoking and the New Health Education in Britain 1950s–1970s’, *American Journal of Public Health*, 2005, 95, 956–64.

27 Central Health Services Council and Scottish Health Services Council, *Health Education* (London: HMSO, 1964), 9.

28 Ian Sutherland, *Health Education—Half a Policy, 1968–86: Rise and Fall of the Health Education Council* (Cambridge: NEC Publications, 1987).

29 The National Archives (hereafter TNA) FP 1/1, The Health Education Council—Questions for the Health Education Council. Submission by the Director General for Consideration on 20 March 1973. Annex A: Extract from Memorandum Dated January 1969: Areas of Concern and Action.

30 DHSS, *Prevention and Health: Everybody’s Business. A Reassessment of Public and Personal Health* (London: HMSO, 1976), 7.

31 TNA MH 154/693, Press Release: Preventive Health Services Can Save Nation Money: Minister. Quality Of Life May Also Improve. 3 November, 1976.

32 Cmnd 7047, *Prevention and Health* (London: HMSO, 1977), 7.

33 Ilona Kickbusch, ‘The Contribution of the World Health Organization to a New Public Health and Health Promotion’, *American Journal of Public Health*, 2003, 93, 383–8.

34 Alex Mold and Virginia Berridge, ‘The History of Health Promotion’, in *Health Promotion Theory*, ed. by Liza Cragg, Maggie Davies & Wendy Macdowall (Maidenhead: Open University Press, 2013), 3–19.

35 Deborah Lupton, *The Imperative of Health: Public Health and the Regulated Body* (London; Thousand Oaks, CA: Sage Publications, 1995), 49–54.

36 Peter Duncan, ‘Failing to Professionalise, Struggling to Specialise: The Rise and Fall of Health Promotion as a Putative Specialism in England, 1980–2000’, *Medical History*, 2013, 57, 377–96; Virginia Berridge and Stuart Blume, *Poor Health: Social Inequality Before and After the Black Report* (London: Routledge, 2003).

37 John Ashton and Howard Seymour, *The New Public Health: The Liverpool Experience* (Open University Press, 1988), 21.

38 Theodore Tulchinsky and Elena Varavikova, ‘What Is the “New Public Health”?’, *Public Health Reviews*, 2010, 32, 25–53.

39 Dorothy Porter, ‘Calculating Health and Social Change: An Essay on Jerry Morris and Late-Modernist Epidemiology’, *International Journal of Epidemiology*, 2007, 36, 1180–84.

40 Niyi Awofeso, ‘What’s New About the “New Public Health”?’, *American Journal of Public Health*, 2004, 94, 705–9.

41 Alan Petersen and Deborah Lupton, *The New Public Health: Health and Self in the age of Risk* (London: Sage, 1996); David Armstrong, ‘Public Health Spaces and the Fabrication of Identity’, *Sociology*, 1993, 27, 393–410.

42 Mariana Valverde, *Diseases of the Will: Alcohol and the Dilemmas of Freedom* (Cambridge: Cambridge University Press, 1998).

43 Brian Harrison, *Drink and the Victorians: The Temperance Question in England 1815–1872*, 2nd edn (Keele: Keele University Press, 1994).

44 Thom, *Dealing with Drink*; Betsy Thom and Virginia Berridge, ‘“Special Units for Common Problems”: The Birth of Alcohol Treatment Units in England’, *Social History of Medicine*, 1995, 8, 75–93.

45 Bill Luckin, ‘A Kind of Consensus on the Roads? Drink Driving Policy in Britain 1945–1970’, *Twentieth Century British History*, 2010, 21, 350–74; Bill Luckin, ‘Anti-Drink Driving Reform in Britain, c. 1920–80’, *Addiction*, 2010, 105, 1538–44.

46 James Nicholls, *The Politics of Alcohol* (Manchester: Manchester University Press, 2009), 204.

47 Royal College of Psychiatrists, *Alcohol: Our Favourite Drug* (London: Tavistock, 1986), 108.

48 Royal College of Physicians, *A Great and Growing Evil: The Medical Consequences of Alcohol Abuse* (London: Tavistock, 1987), 24.

49 Thom, *Dealing with Drink*, 110–20.

50 Lewis, *What Price Community Medicine?*; Gorsky, ‘Local Leadership in Public Health’; John Welshman, ‘The Medical Officer of Health in England and Wales, 1900–1974: Watchdog or Lapdog?’, *Journal of Public Health Medicine*, 19 (1997), 443–50.

51 Martin Gorsky, Karen Lock and Sue Hogarth, ‘Public Health and English Local Government: Historical Perspectives on the Impact of “Returning Home”’, *Journal of Public Health*, 2014, 1–6.

52 Berridge, *Marketing Health*.

53 Central Policy Review Staff, *Alcohol Policies in the UK: The Report of the Central Policy Review Staff* (Stockholm: Sociologiska Institutionen, 1982).

54 Rob Baggott, *Alcohol, Politics and Social Policy* (Aldershot: Avebury, 1990), 74.

55 TNA FP 1/1, Report by the Objectives Committee, 29 October 1973.

56 TNA FP 1/2, Pilot Campaign O Alcoholism, For Consideration, 7 May 1974.

57 TNA FP 1/1, Services To Local Authorities: Submission By The Director General For Consideration on 8 May 1973.

58 Budd, Gray and McCron, *The Tyne Tees Alcohol Education Campaign*; Anthony Thorley, ‘The Role of Mass Communication in Alcohol Health Education’, in Nick Heather, Ian Robertson, and Phil Davies (eds), *The Misuse of Alcohol: Crucial Issues in Dependence Treatment and Prevention* (London: Croom Helm, 1985), 255–75.

59 Cust, ‘Health Education about Alcohol in the Tyne Tees Area’.

60 TNA FP 1/2, Pilot Campaign On Alcoholism—Provisional Budget, 7 May 1974.

61 Alison Fendley, *Saatchi & Saatchi: The Inside Story* (New York: Skyhorse Publishing, Inc., 2011).

62 Berridge and Loughlin, ‘Smoking and the New Health Education’.

63 Cust, ‘Health Education about Alcohol in the Tyne Tees Area’.

64 Budd, Gray and McCron, *The Tyne Tees Alcohol Education Campaign*, 31.

65 Thorley, 261.

66 Budd, Gray and McCron, *The Tyne Tees Alcohol Education Campaign*, 36.

67 TNA FP ½, Meeting of the HEC Council, 19 November 1974.

68 Budd, Gray and McCron, *The Tyne Tees Alcohol Education Campaign*; Thorley.

69 Cust, ‘Health Education about Alcohol in the Tyne Tees Area’.

70 Budd, Gray and McCron, *The Tyne Tees Alcohol Education Campaign*, 41.

71
*Ibid.*, 32.

72 Thorley, ‘The Role of Mass Communication’, 262.

73
*Ibid*.

74 Berridge, *Marketing Health*, 187–94.

75 Budd, Gray and McCron, *The Tyne Tees Alcohol Education Campaign*, 35.

76 Thorley, ‘The Role of Mass Communication’, 263.

77 Budd, Gray and McCron, *The Tyne Tees Alcohol Education Campaign*, 36.

78
*Ibid.*, 39.

79
*Ibid*., 67.

80 Thorley, ‘The Role of Mass Communication’, 266.

81 Royal College of Psychiatrists, *Alcohol and Alcoholism* (London: Tavistock, 1979), 139–40.

82 Budd, Gray and McCron, *The Tyne Tees Alcohol Education Campaign*, 41.

83
*Ibid*., 148–9.

84
*Ibid*., 154.

85 TNA JA 384/1, Points from the Speech of the Secretary of State for Social Services at the Annual General Meeting of the National Council on Alcoholism on 21 July 1981.

86 TNA JA 384/1, HEC, Alcohol Education Programme: Strategy And Proposals for 1982–3.

87 Thom, *Dealing with Drink*, 120–1.

88 Advisory Committee on Alcoholism, 4.

89 TNA MH 154/692, Mrs P.A. Lee to Mr Woodlock: Prevention of Alcoholism. July 1975.

90 TNA MH 154/692, Alcoholism Advisory Committee: Sub-Group on Preventive Strategies, Minutes of the first meeting on 29 January 1976.

91 Advisory Committee on Alcoholism, 8.

92
*Ibid*., 11.

93 TNA MH 154/692, Alcoholism Advisory Committee Sub Group on Prevention: Minutes of meeting on 26 May 1976.

94 TNA MH 154/692, Alcoholism Advisory Committee: Sub-Group on Preventive Strategies, Minutes of the meeting on 17 March 1976.

95 TNA MH 154/692, Letter from Mrs P Lee [DHSS] to ACL Mackie, HEC, 12 April 1976.

96 Advisory Committee on Alcoholism, 6.

97 TNA MH 154/693, ACA Sub-Group On Prevention: Note Of A Discussion On Papers On Fiscal Policy And Alcohol Consumption And On The Ledermann Model Of Alcohol Consumption February 1977.

98 Advisory Committee on Alcoholism, 11.

99 DHSS, *Prevention and Health: Everybody’s Business*, 69.

100 Colin Welch, ‘No Meddles for Ennals’, *Daily Telegraph*, 27 June 1977.

101 Peter Simple, ‘Warning’, *Daily Telegraph*, 11 November 1977.

102 Anon, ‘Alcoholism: Problem Drinkers, Drinking Problems’, *The Economist*, 12 November 1977, 27.

103 TNA MH 154/1124, Press Release: Government To Launch Nationwide Debate On Alcohol Abuse, 7 November 1977.

104 Cmnd 7047, 45.

105 TNA MH 154/693, A. Yarrow to Mr Benham, 29 October 1976. Emphasis in the original.

106 TNA MH 154/1139, Preventive [sic] and Health: Consultative Document on Alcohol (Draft February 1978).

107 TNA MH 154/1141, Letter from D.J. Howard, HM Customs & Excise to Mrs Pearson, DHSS, 3 July 1979.

108 TNA MH 154/1141, Letter from A.J. Gray, Department of Trade to Mr Budd, DHSS, 19 May 1979.

109 TNA MH 154/1528, Consultative Document on Alcohol Misuse: Meeting with MAFF on 6 February 1981.

110 TNA MH 154/1141, Letter from H.M. Griffiths, Treasury to Mrs MAJ Pearson, DHSS, 1 June 1979.

111 TNA MH 154/1531, Letter from Norman Fowler to William Whitelaw, 8 November 1981. See also Thom, *Dealing with Drink*, 119–20.

112 DHSS, *Drinking Sensibly*, 51–3.

113
*Ibid*., 58–9.

114
*Ibid*., 6.

115 TNA MH 154/1137, K.J. Moyes to Mrs Pearson, Prevention and Alcohol, 29 July 1977.

116 TNA MH 154/1531, Mrs M.A.J. Pearson to Mr Warren, printing and stationery, 2 November 1981.

117 Anon, ‘Drive to Halt Big Rise in Heavy Drinking’, *The Guardian*, 15 December 1981.

118 Thomas Bewley, ‘Government Reports: Published and Pirated’, *British Medical Journal*, 285 (1981, 28 August–4 September) 638–40.

119 TNA MH 154/1530, Michael Brown to Mrs Pearson, 20 August 1981.

120 TNA MH 154/1530, Consultative Document on Prevention of Alcohol Misuse: in the Health Department's Prevention and Health Series, no date [December 1979].

121 DHSS, *Drinking Sensibly*, 7.

122
*Ibid*., 8.

123 Health Education Council, *That’s the Limit. Alcohol Information Pamphlet* (London: Health Education Council, 1984).

124 Royal College of Physicians; Royal College of General Practitioners, *Alcohol: A Balanced View* (London: Royal College of General Practitioners, 1987); Royal College of Psychiatrists, *Alcohol: Our Favourite Drug*.

125 Sarah Boseley, ‘Experts Call for Warnings on Alcohol as Men Refuse to Believe Risks’, *The Guardian*, 13 May 2016. <http://www.theguardian.com/society/2016/may/13/warnings-alcohol-middle-age-men-refuse-believe-risks>, accessed 3 June 2016.

126 Jonathan Gornall, ‘Under the Influence: 1. False Dawn for Minimum Unit Pricing’, *BMJ*, 2014, 348.

127 Paul Bennett, Simon Murphy and Christopher Smith, ‘Health Promotion and Alcohol: Some Sober Reflections’, *Health Education Journal*, 1990, 49, 80–4; Peter Anderson, Dan Chisholm and Daniela C Fuhr, ‘Effectiveness and Cost-Effectiveness of Policies and Programmes to Reduce the Harm Caused by Alcohol’, *The Lancet*, 2009, 373, 2234–46.

